# Cost‐effectiveness of neoadjuvant FOLFIRINOX versus gemcitabine plus nab‐paclitaxel in borderline resectable/locally advanced pancreatic cancer patients

**DOI:** 10.1002/cnr2.1565

**Published:** 2022-02-05

**Authors:** Myles A. Ingram, Brianna N. Lauren, Yoanna Pumpalova, Jiheum Park, Francesca Lim, Susan E. Bates, Fay Kastrinos, Gulam A. Manji, Chung Yin Kong, Chin Hur

**Affiliations:** ^1^ Division of General Medicine Columbia University Irving Medical Cancer and the Vagelos College of Physicians and Surgeons New York New York USA; ^2^ Department of Medicine, Vagelos College of Physicians and Surgeons Columbia University New York New York USA; ^3^ Herbert Irving Comprehensive Cancer Center Columbia University Irving Medical Center New York New York USA; ^4^ Division of Digestive and Liver Diseases Columbia University Irving Medical Cancer and the Vagelos College of Physicians and Surgeons New York New York USA; ^5^ Division of General Medicine Mount Sinai School of Medicine New York New York USA

**Keywords:** chemotherapy, clinical cancer research, pancreatic cancer

## Abstract

**Background:**

The 2020 National Comprehensive Cancer Network guidelines recommend neoadjuvant FOLFIRINOX or neoadjuvant gemcitabine plus nab‐paclitaxel (G‐nP) for borderline resectable/locally advanced pancreatic ductal adenocarcinoma (BR/LA PDAC).

**Aim:**

The purpose of our study was to compare treatment outcomes, toxicity profiles, costs, and quality‐of‐life measures between these two treatments to further inform clinical decision‐making.

**Methods and Results:**

We developed a decision‐analytic mathematical model to compare the total cost and health outcomes of neoadjuvant FOLFIRINOX against G‐nP over 12 years. The model inputs were estimated using clinical trial data and published literature. The primary endpoint was incremental cost‐effectiveness ratios (ICERs) with a willingness‐to‐pay threshold of $100 000 per quality‐adjusted‐life‐year (QALY). Secondary endpoints included overall (OS) and progression‐free survival (PFS), total cost of care, QALYs, PDAC resection rate, and monthly treatment‐related adverse events (TRAE) costs (USD). FOLFIRINOX was the cost‐effective strategy, with an ICER of $60856.47 per QALY when compared to G‐nP. G‐nP had an ICER of $44639.71 per QALY when compared to natural history. For clinical outcomes, more patients underwent an “R0” resection with FOLFIRINOX compared to G‐nP (84.9 vs. 81.0%), but FOLFIRINOX had higher TRAE costs than G‐nP ($10905.19 vs. $4894.11). A one‐way sensitivity analysis found that the ICER of FOLFIRINOX exceeded the threshold when TRAE costs were higher or PDAC recurrence rates were lower.

**Conclusion:**

Our modeling analysis suggests that FOLFIRNOX is the cost‐effective treatment compared to G‐nP for BR/LA PDAC despite having a higher cost of total care due to TRAE costs. Trial data with sufficient follow‐up are needed to confirm our findings.

## 1 BACKGROUND

Adoption of neoadjuvant chemotherapy for patients with borderline resectable/locally advanced (BR/LA) pancreatic ductal adenocarcinoma (PDAC) treatment is quickly increasing, and the field is shifting away from adjuvant therapy. Several clinical trials have evaluated the effectiveness of therapies such as FOLFIRINOX and gemcitabine plus nab‐paclitaxel (G‐nP) for PDAC patients.^1^, ^2^, ^3^, ^4^ These regimens have also been used in patients with BR/LA PDAC to downsize tumors to the point where they may be resected. Currently, the 2020 National Comprehensive Cancer Network (NCCN) guidelines recommend either neoadjuvant G‐nP or neoadjuvant FOLFIRINOX for patients with BR/LA PDAC.^5^

In a previous study, we compared the effectiveness and cost‐effectiveness of neoadjuvant versus adjuvant therapies for BR/LA PDAC.^6^ Neoadjuvant FOLFIRINOX was compared to adjuvant gemcitabine and adjuvant gemcitabine plus capecitabine. We found that neoadjuvant. FOLFIRINOX was the optimal strategy when compared to the two adjuvant strategies but were not able to incorporate other neoadjuvant therapies due to a lack of data.

To date, the efficacy and cost‐effectiveness of neoadjuvant FOLFIRINOX and G‐nP in BR/LA PDAC have not been compared in a randomized, prospective clinical trial. Because of this lack of clinical trial data, we developed a decision‐analytic model incorporating the best available published data to simulate a hypothetical clinical trial between FOLFIRNOX and G‐nP. The aim of this study is to compare these two neoadjuvant strategies in terms of cost‐effectiveness for BR/LA PDAC treatment.

## 2 METHODS

### 2.1 Model overview and target population

**FIGURE 1:**
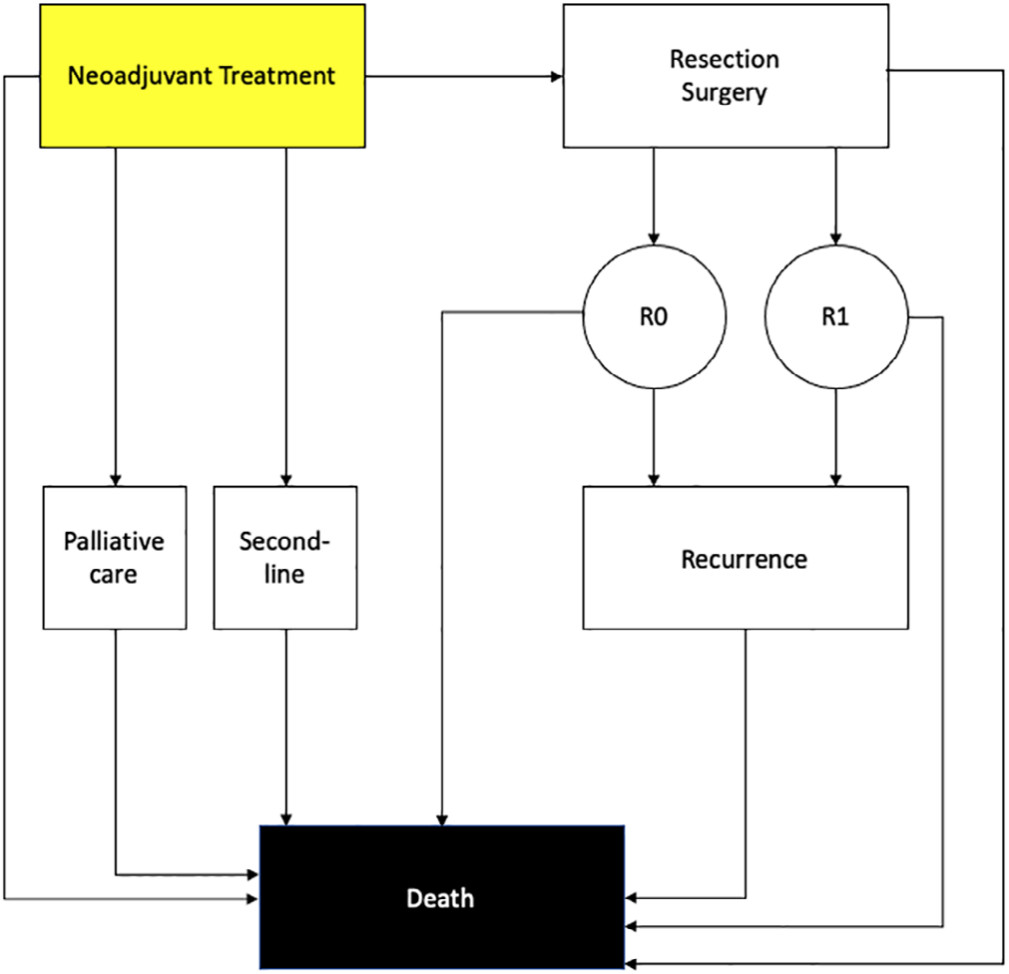
Model schematic. Boxes represent health states, circles represent temporary transitional states. The black death state is absorbing. Arrows denote transitions

We developed a Markov model, using Python 3.7, to follow and track hypothetical cohorts of BR/LA PDAC patients undergoing neoadjuvant therapy prior to resection through a simulated trial (Figure 1). The model cohort consisted of 60‐year‐old BR/LA PDAC patients who have not previously received treatment (first‐line); the model was cycled monthly for 12 years. For this analysis, we assumed a uniform tumor staging and location for each cohort in each strategy in order to have an unbiased comparison between strategies. In terms of tumor staging and location, G‐nP patients and FOLFIRINOX patients had similar characteristics (Table S1). The time between diagnosis (beginning of the model) and resection was 6 months. Patients in the FOLFIRONOX arm received 6 cycles on a day 1 and 15 infusion 28‐day schedule and patients in the G‐nP arm received 6 cycles on a day 1, 8, and 15 infusion 28‐day schedule, consistent with clinical trials and published literature.^7^, ^8^, ^9^, ^10^, ^11^, ^12^, ^13^, ^14^, ^15^, ^16^, ^17^, ^18^, ^19^, ^20^, ^21^, ^22^, ^23^, ^24^, ^25^, ^26^ Causes of death in the model included all‐cause, cancer, and surgical mortality. After the completion of the chemotherapy cycles, patient in the neoadjuvant treatment state, move into the resection state where they receive resection surgery. After resection, patients were assigned R0/R1 status and entered either a remission stage or a recurrence stage. R0/R1 status was defined as whether patients had microscopic cancer cells present at the margin of the primary tumor after resection. From the remission stage, patients either transitioned to the recurrence stage or died from all causes. From the recurrence stage, patients either died from cancer or all cause. All patients who did not complete neoadjuvant therapy received either palliative care, second‐line treatment, or died. Additionally, we factored in both the likelihood that a patient would develop adverse events during neoadjuvant chemotherapy and the probability of resection surgery complications. Patients in the natural history arm received no neoadjuvant treatment or surgery for their BR/LA PDAC.

Furthermore, we included a hypothetical scenario analyses to our model. This scenario explored the base analysis in a center of excellence setting. For this scenario, the dropout rates, PDAC recurrence rates, and R0 rates were adjusted to align with published literature from centers of excellence.^27^, ^28^, ^29^ We defined a center of excellence as a highly specialized and interdisciplinary program within a healthcare institution that supplies expertise and resources to a particular medical area, in this case PDAC. We included the hypothetical scenario analysis to investigate what effect a center‐of‐excellence setting had on our model results. The parameters of the scenario can be found in Table S2.

### 2.2 Study perspective and outcomes

We assessed the incremental cost‐effectiveness of G‐nP versus FOLFIRINOX from the perspective of the U.S. healthcare system. The primary endpoint was the optimal neoadjuvant treatment, defined as the highest QALYs with an incremental cost‐effectiveness ratio (ICER) below a willingness‐to‐pay threshold (WTP) of $100 000 (2021 USD). Secondary endpoints included overall survival (OS), progression‐free survival (PFS), total cost of care, R0 status, PDAC resection rate, and monthly treatment‐related adverse events (TRAE) costs. Costs and QALYs were discounted at 3% per year, and a half‐cycle correction was applied to QALYs and unadjusted life‐years.

### 2.3 Health state transition probabilities, model calibration

We estimated the transition probabilities of our Markov model from published literature and clinical trial data (Tables 1 and 2). We calibrated our model by fitting the overall and progression free survival curves of each arm, including the natural history arm, to published Kaplan–Meier curves.^7^, ^8^, ^9^, ^10^, ^11^, ^32^ We extracted data from retrospective published data and preliminary clinical trial data to inform our model. In instances where BR/LA PDAC neoadjuvant treatment data was limited, we used conservative estimates from metastatic patient cohorts. We used a software called Engauge Digitizer to extract the data from the overall and progression free survival curves from published literature and used that data to calibrate cancer progression rates of our model. FOLFIRINOX clinical trials were more inclined to have younger patients, lower Charlson Comorbidity Index (CCI) scores, and higher likelihood of patients with N0 status than G‐nP clinical trials. Since FOLFIRINOX clinical trials tend to enroll healthier and more robust patients, we used a more conservative estimate of overall survival and progression‐free survival in the FOLFIRINOX trials to reconcile the baseline differences that like existed between patients enrolled in FOLFIRINOX and G‐nP trials (Table S1). We also validated our model by comparing model outputs such as R0 rate, patient resection rate, and median OS/PFS with published clinical endpoints.^6^, ^7^, ^8^, ^9^, ^10^, ^30^, ^31^, ^32^, ^33^, ^34^, ^35^, ^36^, ^37^, ^38^, ^39^, ^40^, ^41^, ^42^ All‐cause mortality was derived from the average of male and female 2016 U.S. life tables.

**TABLE 1:** General model parameters

General parameters			
Parameters	Value	Range for sensitivity analysis	Source
Age (years)	60	38–66	6,7,30,31
30‐day BR/LA PDAC mortality rate	0.24	0.17–0.57	6,17,30
30‐day surgical mortality rate	0.015	0.01–0.053	6,32
Post‐surgical pancreatic fistula rate	0.093	0–0.24	6,33,34
Progression‐free survival BR/LA PDAC	0.8	0.68–0.88	6,23,24,27
*Costs*			
PDAC resection surgery cost	$29580.00	$15000.00–$41000.00	6,35
Palliative care cost	$101388.00	$92820.00–$103020.00	6,36
Capecitabine and radiation per month	$1377.00	$840.00–$1938.00	6,15
Chemoradiation hospitalization costs	$2856.00	$1734.00–$3774.00	6,15
Endoscopic Ultrasound	$1570.80	$0.00–$1570.80	6,27
PDAC costs per month (inpatient)	$5508.00	$3162.00–$7446.00	6,22
*Utilities*			
Progression‐free PDAC utility	0.80	0.68–0.88	6,17,23,24,27
Progressive disease	0.73	0.62–0.80	6,17,23,24,27
Palliative care	0.14	0–0.34	6,23
Recovery from surgery	0.78	0.78–0.81	6,27

Abbreviations: BR/LA, Borderline resectable/locally advanced; PDAC, pancreatic ductal adenocarcinoma.

**TABLE 2:** Strategy specific parameters

**FOLFIRINOX parameters**			
Chemotherapy cycle length (months)	6		7,37
Dropout rate	0.35	0.33–0.4	7,32,38
Toxicity rate	0.75	0.287–0.85	7,13,31,39
Surgical complication rate	0.36	0.29–0.43	7,30
Post‐surgical pancreatic fistula rate	0.05	0–0.05	7,37
R0 rate	0.85	0.40–0.88	6,7,37
PDAC recurrence rate	0.61	0.29–0.65	6,7,37
Hospitalization for toxicity	0.37	0.26–0.46	6,7,37
Lymph node positivity	0.56	0.50–0.62	7,37
Survival after recurrence (R0 resection) (months)	21	19–23	7,37,40
Survival after recurrence (R1 resection) (months)	17	15–19	7,37,40
Survival after recurrence (N0 disease) (months)	22	20–24	7,37,40
Survival after recurrence (N1 disease) (months)	18	16–20	7,37,40
Survival on second‐line therapy (months)	9	7–11	7,9,41
*Costs*			
First‐line chemotherapy costs per cycle	$863.50	$760.00–$960.00	6
Toxicity costs per cycle (first‐line)	$1734.00	$1387.20–$2080.80	17–19
Second‐line therapy costs per month	$13209.00	$0.00–$14800.00	21
Toxicity costs per month (second‐line)	$6778.80	$5423.04–$9490.32	16
Administration cost per month (first‐line and second‐line)	$578.79	$450.00–$700.00	6
*Utilities*			
Chemotherapy disutility	−0.12	−0.19–0.05	6,17
Chemotherapy toxicity disutility	−0.24	−0.28–0.19	6,17,25,42
**Nab‐paclitaxel plus gemcitabine parameters**			
Chemotherapy cycle length (months)	6		6,7
Dropout rate	0.31	0.14–0.39	7,11,32
Toxicity rate	0.65	0.15–0.75	7,11,32
Complete cycles	6		7,11,32
Surgical complication rate	0.23	0.15–0.4	7,11,32
Post‐surgical pancreatic fistula rate	0	0–0.015	7,11,32
R0 rate	0.81	0.44–0.88	6,7,11,32
PDAC recurrence rate	0.53	0.29–0.65	6,7,11,32
Hospitalization for toxicity	0.25	0.15–0.35	6,7,11,32
Lymph node positivity	0.72	0.71–0.86	6,7
Survival after recurrence (R0 resection) (months)	18.7	16–21	6,7
Survival after recurrence (R1 resection) (months)	16	14–18	6,7
Survival after recurrence (N0 disease) (months)	20	18–22	6,7
Survival after recurrence (N1 disease) (months)	16	14–18	6,7
Survival on second‐line therapy (months)	9	7–11	7,9
*Costs*			
First‐line chemotherapy cost per cycle	$8882.41	$8000 $10 000	6,26
Toxicity cost per cycle (first‐line)	$918.00	$734.40 $1101.60	6,26
Second‐line chemotherapy cost per month	$4080.00	$3000.00–$4800.00	6,21
Toxicity cost per month (second‐line)	$2094.75	$1675.80–$2513.70	6,26
Administration cost per month (first‐line and second‐line)	$568.98	$450–$700	6
*Utilities*			
Chemotherapy disutility	−0.041	−0.071–0.031	6,26
Chemotherapy toxicity disutility	−0.10	−0.11–0.091	6,26

Abbreviations: BR/LA, Borderline resectable/locally advanced; PDAC, pancreatic ductal adenocarcinoma.

### 2.4 Costs and health state utility values

Costs were calculated from a payer perspective and no indirect costs were included in the analysis. The costs of chemotherapy drugs were based on Centers for Medicare and Medicaid (CMS) 2020 average sale price. Capecitabine and radiation therapy costs for the neoadjuvant arms were estimated from published literature.^6^, ^30^ Costs associated with hospitalization for TRAE were based on Medicare reimbursement rates from the CMS Physician Fee Schedule. Rates of hospitalization due to TRAE were estimated from clinical trial data and published literature. Costs associated with PDAC treatment, palliative care, second‐line treatment, drug administration, and resection surgery were derived from Medicare reimbursement rates used in previous cost‐effectiveness analyses of neoadjuvant PDAC treatment.^12^ ^,^ ^31^, ^32^, ^33^, ^34^, ^35^, ^36^ Additionally, all utilities and dis‐utilities used to calculate QALYs were based on previously published cost‐effectiveness analyses.^12^, ^13^, ^37^, ^38^, ^39^, ^40^, ^41^ All costs were inflation‐adjusted to 2021. References for utility and cost estimates can be found in Tables 1 and 2.

### 2.5 Sensitivity analyses

We performed one‐way deterministic sensitivity analyses, which involved changing each parameter individually across a plausible range of values, to determine the robustness of our base‐case results (Tables 1 and 2). Probabilistic sensitivity analyses were also conducted by changing all input parameters at the same time. We sampled the parameter values on specific distributions and ran 10 000 iterations to investigate how ICER of the optimal strategy was affected.

## 3 RESULTS

### 3.1 Base‐case analyses

**FIGURE 2:**
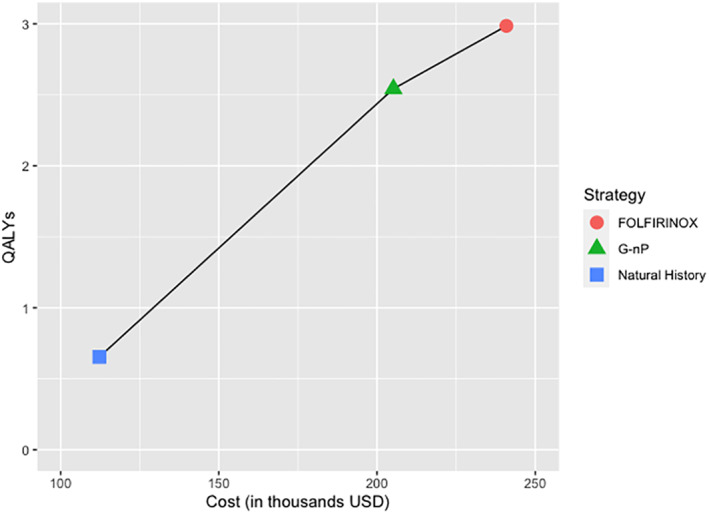
Base case efficiency frontier. G‐nP, Gemcitabine plus nab‐paclitaxel; QALYs, quality‐adjusted life‐years

Neoadjuvant FOLFIRINOX treatment was the optimal strategy in the base‐case model with a total cost of $240 877 and 2.99 QALYs. Furthermore, patients in this arm had a resection rate of 67.32%, with 84.90% of those undergoing surgery achieving an R0 resection. Patients in the FOLFIRINOX arm had TRAE costs of $10 905 per month. FOLFIRINOX yielded an ICER of $80 862 compared to neoadjuvant G‐nP treatment, which was below the $100 000 WTP threshold. G‐nP had a total cost of $205 161, 2.54 QALYs, and an ICER of $49 196 compared to natural history (Figure 2). Patients in the G‐nP arm had a resection rate of 59.33%, with 80.99% of patients undergoing surgery achieving an R0 resection (Table S3). The G‐nP arm was associated with TRAE costs of $4894 per month. In terms of survival, FOLFIRINOX had a higher median OS and PFS and higher 10‐year OS and PFS compared to G‐nP (median OS 34.01 vs. 28.27 months; median PFS 29.54 vs. 24.88; 10‐year OS 9.73% vs. 5.24%; 10‐year PFS 8.48% vs. 4.38%; Figure S1). The natural history arm had a median OS/PFS of 12.13/7.29 months and no survival after 10 years (Table 3).

**TABLE 3:** Base‐case results

	Life‐years	Cost (USD)	QALYs	Median OS/PFS (months)	5‐year OS/PFS	10‐year OS/PFS	TRAE cost per month	R0 resection^a^	ICERs
Natural history	1.16	$112 251	0.654	13/7.87	0.20/0.01%	0/0%	–	–	–
G‐nP	3.41	$205 161	2.54	28.27/24.88	21.33/17.92%	5.24/4.38%	$4894	80.99%	$49 196
FOLFIRINOX	4.07	$240 877	2.99	34.01/29.54	29.38/25.68%	9.73/8.48%	$10 905	84.90%	$80 862

^a^ R0 resection % only includes those who made it to surgery.

### 3.2 Center‐of‐excellence scenario

In the center‐of‐excellence scenario, FOLFIRINOX remained the optimal strategy. In this scenario, FOLFIRINOX had an ICER of $73 124, total cost of $251 718, and 3.42 QALYs. The G‐nP strategy resulted in an ICER of $45 795, total cost of $217 753, 2.96 QALYs. Both FOLFIRINOX and G‐nP had higher median, 5‐year, and 10‐year OS and PFS in this scenario compared to the base case, but a smaller percentage of the cohort underwent resection compared to the base case scenario (FOLFIRINOX 57.9 vs. 67.32%; G‐nP 52.6 vs. 59.33%; Table S4).

### 3.3 Sensitivity analyses

The model outcomes were most sensitive to monthly toxicity cost for treatment, resection surgery cost, PDAC recurrence rate, PDAC survival rate, and rate of TRAEs. Increasing monthly toxicity costs for FOLFIRINOX caused the strategy to exceed the $100 000 WTP threshold, thus making G‐nP the optimal strategy. Increasing the rate of TRAEs, the dropout rate, and the PDAC recurrence rate for FOLFIRINOX all resulted in an ICER above the WTP threshold and decreasing the PDAC progression‐free survival rate caused G‐nP to dominate FOLFIRINOX (Figure S2). For G‐nP, the ICER did not exceed the $100 000 WTP threshold for all the plausible ranges of input parameters tested (Figure S3). Additionally, although the model was also sensitive to R0 rate, PDAC mortality rate, second‐line treatment cost, and utility associated with progressive disease, changing these parameters within the pre‐specified parameter ranges did not alter the model results.

The probabilistic sensitivity analysis results demonstrated that the base‐case results were robust to parameter uncertainty. FOLFIRINOX remained the optimal strategy in 76.8% of the simulations, while G‐nP was optimal in the remaining 23.2% with a WTP threshold of $100 000 (Figure S4). The FOLFIRINOX strategy was the cost‐effective strategy 37.2% of the simulations with a WTP threshold of $50 000 and 89.7% of the time with a WTP threshold of $150 000 (Figure S5).

## 4 DISCUSSION

The results of our analysis found that neoadjuvant FOLFIRINOX is the preferred treatment strategy for patients with BR/LA PDAC compared to neoadjuvant G‐nP by multiple endpoints. Although FOLFIRINOX had the highest monthly TRAE cost, it was the cost‐effective strategy as the cost to gain a QALY was below the defined threshold. The strategy also yielded superior OS, PFS, QALYs, and R0 resection rates in our modeling projections.

As we described in the methods, we included a hypothetical scenario analysis to explore how the results of the base case changed in a center‐of‐excellence setting. In the hypothetical scenario where patients were only treated at a center‐of‐excellence, FOLFIRINOX remained the optimal treatment strategy compared to Gn‐P. For the center‐of‐excellence analysis, we found that although fewer patients underwent resection (FOLFIRINOX 57.9 vs. 67.32%; G‐nP 52.6 vs. 59.33%), and that overall survival was longer in this scenario compared to the FOLFIRINOX arm in the base‐case analysis. This result may be explained by a sicker patient population at the center‐of‐excellence, but improved surgical outcomes compared to patients treated in centers without such a designation.^27^, ^42^ Future clinical trials are necessary to confirm our results for this hypothetical model.

To the best of our knowledge, this is the first study to directly compare the cost‐effectiveness of neoadjuvant FOLFIRINOX and neoadjuvant G‐nP for BR/LA PDAC patients. Our previous study compared the cost‐effectiveness of neoadjuvant FOLFIRINOX to adjuvant therapies, but did not include a comparator neoadjuvant treatment arm. Additionally, this study is the first to analyze the cost‐effectiveness of neoadjuvant BR/LA PDAC treatment strategies in a center‐of‐excellence setting, albeit through a “thought experiment.” Simulation modeling analysis enables comparisons that would be difficult or impossible to evaluate in a clinical trial. These outcomes include 10‐year overall survival and progression‐free survival, QALYs, and costs. In the future, additional clinical trial data will be incorporated into the model to update the analyses to the latest PDAC findings.

Our results must be interpreted with study limitations in mind. Due to the scarcity of prospective, randomized clinical trial data for patients with BR/LA PDAC treated with neoadjuvant chemotherapies, model assumptions and inputs relied on preliminary clinical trial data and retrospective published data. Using these data sources may have incorporated bias in our analysis, including referral bias, and/or selection bias of healthier patients compared to the average BR/LA PDAC patient. In instances where BR/LA data was limited, we consistently used the more conservative estimates from metastatic patient cohorts, biasing our results against neoadjuvant FOLFIRINOX or the null. Additionally, FOLFIRINOX trials have a selection bias, often enrolling healthier patients compared to G‐nP trials. We attempted to reconcile this selection bias by calibrating to a more conservative estimate of the overall survival and progression‐free survival confidence interval in FOLFIRINOX trials so that G‐nP and FOLFIRINOX could be compared with minimal bias. When there was limited cost and utility data for the neoadjuvant setting, we used published data from metastatic PDAC cohorts to derive conservative estimations. Lastly, we also assumed uniform comorbidity status across patients assigned to all treatment arms in the model, which is likely an oversimplification of real‐life clinical practice. Given high rates of treatment related toxicity, comorbidities and patient performance status play an important role in individualizing a neoadjuvant treatment plan for patients with BR/LA PDAC. Because of these data limitations, we assuaged concerns of uncertainty about our model by verifying our model outputs through rigorous sensitivity analyses and aligning our outcomes with published clinical data.

## 5 CONCLUSION

In summary, our analysis found that FOLFIRNOX is the optimal neoadjuvant chemotherapy regimen compared to gemcitabine plus nab‐paclitaxel for BR/LA PDAC, despite having a higher cost of total care due to TRAE costs. This comparison of neoadjuvant PDAC chemotherapy strategies gives new insight into the selection of treatment regimens for BR/LA patients. Data from prospective randomized clinical trials with sufficient follow‐up are needed to confirm our findings.

## CONFLICT OF INTEREST

The authors declare no conflict of interest.

## AUTHOR CONTRIBUTIONS

*Study concept and design, acquisition of data, analysis and interpretation of data, drafting of the manuscript, statistical analysis, technical support*, M.A.I.; *Study concept and design, acquisition of data, analysis and interpretation of data, drafting of the manuscript, statistical analysis, technical support*, B.L.; *Acquisition of data, analysis and interpretation of data, drafting of the manuscript*, Y.P.; *Acquisition of data, analysis and interpretation of data, critical revision of manuscript for important intellectual content*, J.P.; *Acquisition of data, analysis and interpretation of data, critical revision of manuscript for important intellectual content*, F.L.; *Analysis and interpretation of data, critical revision of the manuscript for important intellectual content, technical support*, S.B.; *Analysis and interpretation of data, revision of the manuscript for important intellectual content*, F.K.; *Critical revision of the manuscript for important intellectual content, analysis and interpretation of data*, G.A.M.; *Critical revision of the manuscript for important intellectual content, analysis and interpretation of data*, C.Y.K.; *Study concept and design, critical revision of the manuscript for important intellectual content, obtained funding, technical and material support, study supervision*, C.H.

## ETHICAL STATEMENT

This study was approved by the Institutional Review Board at Columbia University Medical Center, New York.

## Supporting information


**Appendix S1**: Supporting Information

## Data Availability

The data that support the findings of this study are available under CUMC‐HIRE in the PDAC_model repository at https://github.com/CUMC-HIRE/PDAC_model
