# Supplementary material for: Cost‐effectiveness of neoadjuvant FOLFIRINOX versus gemcitabine plus nab‐paclitaxel in borderline resectable/locally advanced pancreatic cancer patients
Source: Cancer Rep (Hoboken). 2022 Feb 5;5(9):e1565. doi: 10.1002/cnr2.1565 (PMC9458514; doi:10.1002/cnr2.1565)
Supplement: Supplementary file 1 — Appendix S1: Supporting Information [file CNR2-5-e1565-s001.docx]

**Supplemental Materials**

**Supplemental Table 1a. Baseline Patient Characteristics from FOLFIRINOX Literature**

| **Parameters** | **Michelakos et al** | **Dhir et al** | **Conroy et al** | **Kim et al** |
| --- | --- | --- | --- | --- |
| Study Size | 141 | 73 | 247 | 22 |
| Median Age | 63 | 63 | 63 | 62.5 |
| CCI | 1 | 4 | 1 | 1 |
| Tumor Size (cm) | 3.1 | 2.9 | 3 | 2 |
| Tumor Location | N/A | N/A | N/A |  |
| Head |  |  |  | 12 (54.5) |
| Uncinate |  |  |  | 5 (22.7) |
| Neck |  |  |  | 2 (9.1) |
| Body/Tail |  |  |  | 3 (13.7) |
| Locally Advanced (%) | 69 (48.9) | 15 (20.9) | 43 (17.4) | N/A |
| Borderline (%) | 72 (51.1) | 58 (79.4) | 183 (74.1) | N/A |
| R0 status | 87 (80.6) | 62 (84.9) | 148 (59.9) | 20 (90.9) |
| N0 status | 40 (36.4) | 32 (43.8) | 59 (23.9) | N/A |
| Lymphatic Invasion | 30 (27.3) | 43 (61.4) | 154 | N/A |

**Supplemental Table 1b. Baseline Patient Characteristics from G-nP Literature**

| **Parameters** | **Dhir et al** | **Reni et al** | **Ielpo et al** |
| --- | --- | --- | --- |
| Study Size | 120 | 28 | 45 |
| Median Age | 69 | 66 | 63 |
| CCI | 5 | 2 | 1 |
| Tumor Size (cm) | 2.7 | 2.4 | 3.5 |
| Tumor Location | N/A |  |  |
| Head |  | 20 (71.4) | 25 (55.6) |
| Uncinate |  | 0 (0) | 7 (15.6) |
| Neck |  | 3 (10.7) | 0 (0) |
| Body/Tail |  | 5 (17.9) | 13 (28.8) |
| Locally Advanced (%) | 49 (40.8) | 13 (46) | 17 (37.8) |
| Borderline (%) | 71 (59.2) | 15 (54) | 28 (62.2) |
| R0 status | 97 (80.8) | 4 (14) | 44 (97.8) |
| N0 status | 34 (28.3) | 2 (7) | 59 (23.9) |
| Lymphatic Invasion | 95 (81.2) | N/A | N/A |

**Supplemental Table 2. Center-of-Excellence Scenario Parameters**

| **General Parameters** | | | | |
| --- | --- | --- | --- | --- |
| **Parameters** | **Value** | | **Source** | |
| Age (years) | 60 | | ^1–4^ | |
| 30-day LA/BR PDAC mortality rate | 0.18 | | ^5–7^ | |
| 30-day Surgical mortality rate | 0.015 | | ^1,8^ | |
| Post-surgical pancreatic fistula rate | 0.093 | | ^1,9,10^ | |
| LA/BR PDAC Progression-free survival rate | 0.88 | | ^5,7,11,12^ | |
| **Costs** | | | | |
| PDAC resection surgery cost | $29,580 | | ^1,13^ | |
| Palliative care cost | $101,388 | | ^1,14^ | |
| Capecitabine and radiation per month | $1,377 | | ^1,15^ | |
| Chemoradiation hospitalization costs | $2,856 | | ^1,15^ | |
| Endoscopic Ultrasound | $1,571 | | ^1,12^ | |
| PDAC costs per month (inpatient) | $5,508 | | ^1,16^ | |
| **Utilities** | | | | |
| Progression-free PDAC utility | 0.80 | | ^1,11,12,17,18^ | |
| Progressive disease | 0.73 | | ^1,11,12,17,18^ | |
| Palliative care | 0.14 | | ^1,11^ | |
| Recovery from surgery | 0.78 | | ^1,12^ | |
| **FOLFIRINOX Parameters** | | | | |
| Chemotherapy cycle length (months) | 6 | | ^2,19^ | |
| Dropout rate | 0.4 | | ^2,5,7,8,20^ | |
| Toxicity rate | 0.75 | | ^2,4,5,7,21,22^ | |
| Surgical complication rate | 0.36 | | ^2,3^ | |
| Post-surgical pancreatic fistula rate | 0.05 | | ^2,19^ | |
| R0 rate | 0.88 | | ^1,2,5,6,19^ | |
| PDAC recurrence rate | 0.43 | | ^1,2,5,6,19^ | |
| Hospitalization for toxicity | 0.37 | | ^1,2,19^ | |
| Lymph node positivity | 0.56 | | ^2,19^ | |
| Survival after recurrence (R0 resection) (months) | 21 | | ^2,5,19,23^ | |
| Survival after recurrence (R1 resection) (months) | 17 | | ^2,19,23^ | |
| Survival after recurrence (N0 disease) (months) | 22 | | ^2,19,23^ | |
| Survival after recurrence (N1 disease) (months) | 18 | | ^2,19,23^ | |
| Survival on second-line therapy (months) | 9 | | ^2,24,25^ | |
| **Costs** | | | | |
| First line chemotherapy cost per cycle | $864 | | ^1^ | |
| Toxicity cost per cycle (first line) | $1,734 | | ^17,26,27^ | |
| Second line chemotherapy cost per month | $13,209 | | ^28^ | |
| Toxicity cost per month (second-line) | $6,779 | | ^29^ | |
| Administration cost per month (first-line and second-line) | $579 | | ^1^ | |
| **Utilities** | | | | |
| Chemotherapy disutility | -0.19 | | ^1,5,6,17^ | |
| Chemotherapy toxicity disutility | -0.28 | | ^1,5,6,17,30,31^ | |
| **Nab-Paclitaxel plus Gemcitabine Parameters** | | | | |
| Chemotherapy cycle length (months) | 6 | ^1,2^ | |  |
| Dropout rate | 0.39 | ^2,5–7,32,33^ | |  |
| Toxicity rate | 0.65 | ^2,5,7,32,33^ | |  |
| Surgical complication rate | 0.23 | ^2,32,33^ | |  |
| Post-surgical pancreatic fistula rate | 0 | ^2,32,33^ | |  |
| R0 rate | 0.88 | ^2,5,6,32,33^ | |  |
| PDAC recurrence rate | 0.65 | ^1,2,5,6,32,33^ | |  |
| Hospitalization for toxicity | 0.25 | ^1,2,32,33^ | |  |
| Lymph node positivity | 0.72 | ^1,2,32,33^ | |  |
| Survival after recurrence (R0 resection) (months) | 21 | ^1,2,5,7^ | |  |
| Survival after recurrence (R1 resection) (months) | 16 | ^1,2^ | |  |
| Survival after recurrence (N0 disease) (months) | 20 | ^1,2^ | |  |
| Survival after recurrence (N1 disease) (months) | 16 | ^1,2^ | |  |
| Survival on second-line therapy (months) | 9 | ^1,2^ | |  |
| **Costs** | | | | |
| First line chemotherapy cost per cycle | $8,882 | ^1,34^ | |  |
| Toxicity cost per cycle (first line) | $918 | ^1,34^ | |  |
| Second line chemotherapy cost per month | $4,080 | ^1,28^ | |  |
| Toxicity cost per month (second line) | $2,095 | ^1,34^ | |  |
| Administration cost per month (first-line and second-line) | $569 | ^1^ | |  |
| **Utilities** | | | | |
| Chemotherapy disutility | -0.071 | ^1,5,34^ | |  |
| Chemotherapy toxicity disutility | -0.11 | ^1,5,34^ | |  |

**BR/LA, Borderline resectable/locally advanced; PDAC, pancreatic ductal adenocarcinoma**

**Supplemental Table 3. Additional Clinical Endpoints**

|  | **5-Year Cancer Death** | **10-Year Cancer Death** | **5-Year**  **All Cause Death** | **10-Year**  **All Cause Death** | **R1 resection*** | **% Receiving**  **Surgery** |
| --- | --- | --- | --- | --- | --- | --- |
| Natural History | 98.71% | 98.91% | 1.09% | 1.09% | -- | -- |
| G-nP | 69.71% | 88.34% | 3.03% | 4.05% | 16.94% | 59.33% |
| FOLFIRINOX | 64.43% | 83.94% | 3.15% | 4.53% | 13.43% | 67.32% |

***R1 resection % only includes those who made it to surgery**

**Supplemental Table 4. Center-of-Excellence Model Results**

|  | **Life-Years** | **Cost (USD)** | **QALYs** | **Median OS/PFS (Months)** | **5 Year OS/PFS** | **10 Year OS/PFS** | **% Receiving Surgery** | **R0 resection*** | **ICERs** |
| --- | --- | --- | --- | --- | --- | --- | --- | --- | --- |
| Natural History | 1.16 | $112,251 | 0.654 | 13/  7.87 | 0.20%/  0.01% | 0%/0% | -- | -- | -- |
| G-nP | 3.96 | $217,753 | 2.96 | 32.85/  26.28 | 28.69%/  24.11% | 10.89%/  9.04% | 52.6% | 81.0% | $45,795 |
| FOLFIRINOX | 4.60 | $251,718 | 3.42 | 36.27/  30.7 | 35.64%/  31.42% | 17.60%/  15.44% | 57.9% | 84.7% | $73,124 |

***R0 resection % only includes those who made it to surgery**

**Supplemental Figure 1a. Overall Survival per Strategy**


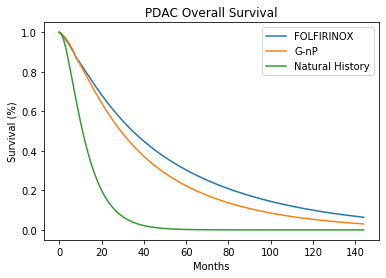


**Supplemental Figure 1b. Progression-Free Survival per Strategy**


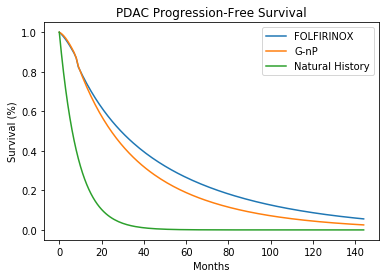


**Supplemental Figure 2a. FOLFIRINOX Cost One-Way Sensitivity Analysis**


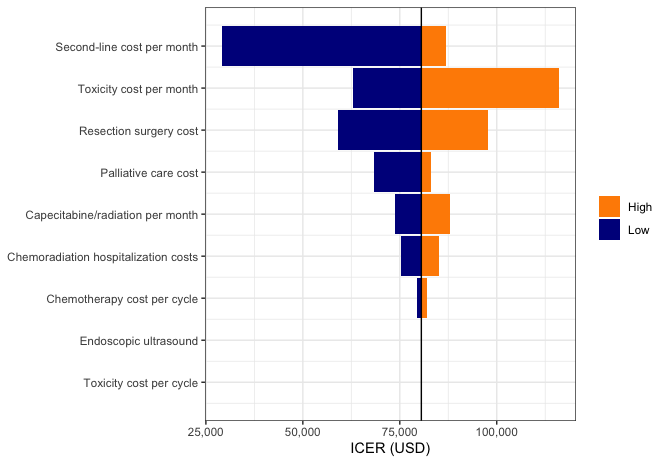


**Supplemental Figure 2b. FOLFIRINOX Utility One-Way Sensitivity Analysis**


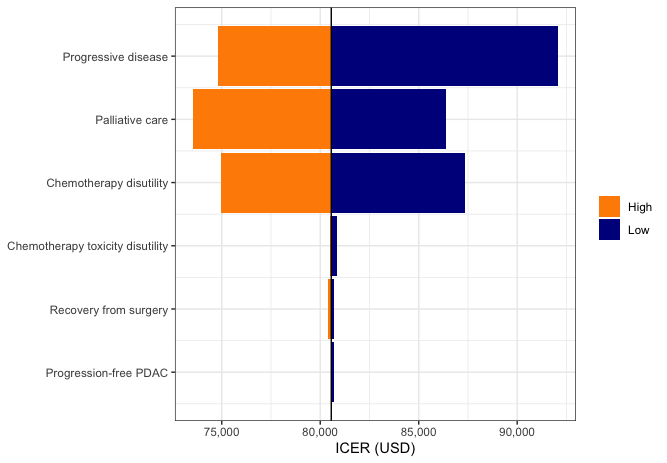


**Supplemental Figure 2c. FOLFIRINOX Probability One-Way Sensitivity Analysis**


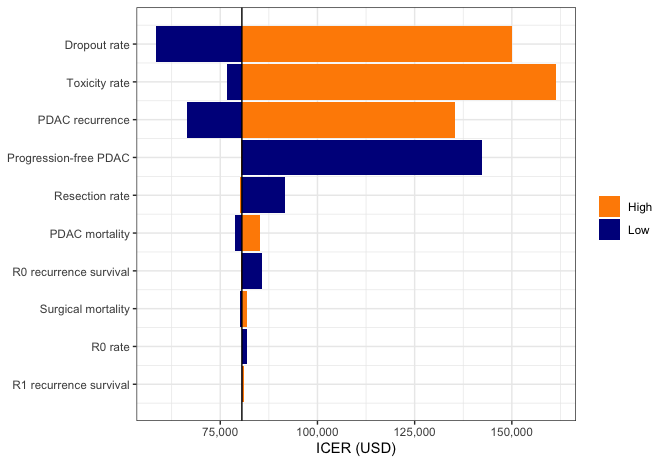


**Supplemental Figure 3a. G-nP Cost One-Way Sensitivity Analysis**


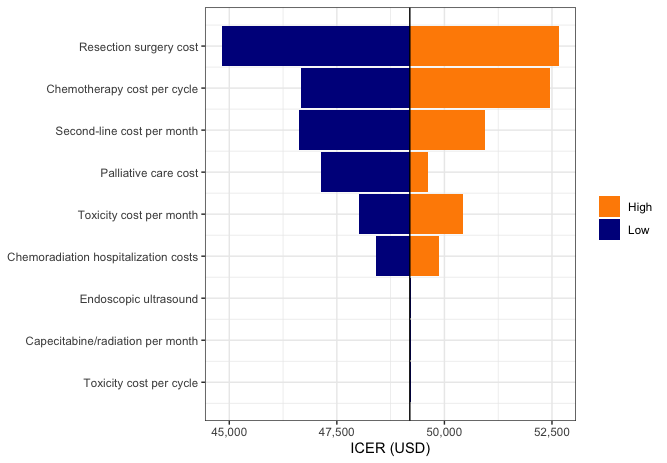


**Supplemental Figure 3b. G-nP Utility One-Way Sensitivity Analysis**


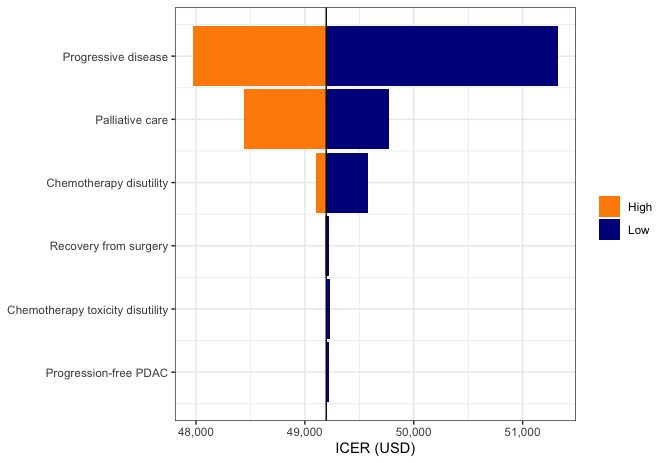


**Supplemental Figure 3c. G-nP Probability One-Way Sensitivity Analysis**


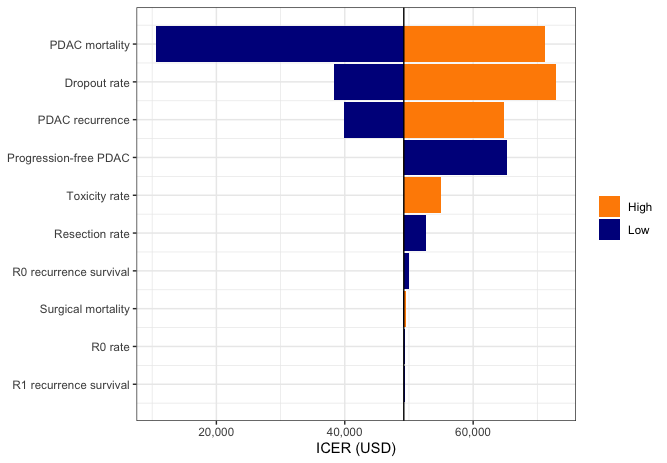


**Supplemental Figure 4. Probabilistic Sensitivity Analysis**


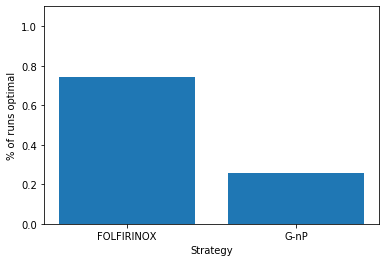


**Supplemental Figure 5. Cost-Effective Acceptability Curve**

**
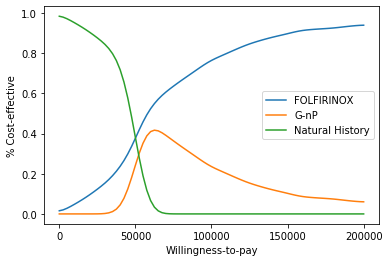
**

**References**

1. Choi, J. G. *et al.* Neoadjuvant FOLFIRINOX for Patients with Borderline Resectable or Locally Advanced Pancreatic Cancer: Results of a Decision Analysis. *The Oncologist* **24**, 945–954 (2019).

2. Dhir, M. *et al.* FOLFIRINOX Versus Gemcitabine/Nab-Paclitaxel for Neoadjuvant Treatment of Resectable and Borderline Resectable Pancreatic Head Adenocarcinoma. *Ann. Surg. Oncol.* **25**, 1896–1903 (2018).

3. Ferrone, C. R. *et al.* Radiological and surgical implications of neoadjuvant treatment with FOLFIRINOX for locally advanced and borderline resectable pancreatic cancer. *Ann. Surg.* **261**, 12–17 (2015).

4. Hosein, P. J. *et al.* A retrospective study of neoadjuvant FOLFIRINOX in unresectable or borderline-resectable locally advanced pancreatic adenocarcinoma. *BMC Cancer* **12**, 199 (2012).

5. Elrod, J. K. & Fortenberry, J. L. Centers of excellence in healthcare institutions: what they are and how to assemble them. *BMC Health Serv. Res.* **17**, 425 (2017).

6. Sherman, W. H. *et al.* Neoadjuvant gemcitabine, docetaxel, and capecitabine followed by gemcitabine and capecitabine/radiation therapy and surgery in locally advanced, unresectable pancreatic adenocarcinoma: Unresectable Locally Advanced Pancreatic Cancer. *Cancer* **121**, 673–680 (2015).

7. Griffin, J. F., Poruk, K. E. & Wolfgang, C. L. Pancreatic cancer surgery: past, present, and future. *Chin. J. Cancer Res. Chung-Kuo Yen Cheng Yen Chiu* **27**, 332–348 (2015).

8. Gillen, S., Schuster, T., Meyer Zum Büschenfelde, C., Friess, H. & Kleeff, J. Preoperative/neoadjuvant therapy in pancreatic cancer: a systematic review and meta-analysis of response and resection percentages. *PLoS Med.* **7**, e1000267 (2010).

9. Alexakis, N., Sutton, R. & Neoptolemos, J. P. Surgical treatment of pancreatic fistula. *Dig. Surg.* **21**, 262–274 (2004).

10. Bassi, C. *et al.* Pancreatic fistula rate after pancreatic resection. The importance of definitions. *Dig. Surg.* **21**, 54–59 (2004).

11. Tam, V. C. *et al.* Cost-effectiveness of systemic therapies for metastatic pancreatic cancer. *Curr. Oncol. Tor. Ont* **20**, e90–e106 (2013).

12. Leung, H. W. C., Chan, A. L. F. & Muo, C.-H. Cost-effectiveness of Gemcitabine Plus Modern Radiotherapy in Locally Advanced Pancreatic Cancer. *Clin. Ther.* **38**, 1174–1183 (2016).

13. Holbrook, R. F., Hargrave, K. & Traverso, L. W. A prospective cost analysis of pancreatoduodenectomy. *Am. J. Surg.* **171**, 508–511 (1996).

14. Doyle, C., Crump, M., Pintilie, M. & Oza, A. M. Does palliative chemotherapy palliate? Evaluation of expectations, outcomes, and costs in women receiving chemotherapy for advanced ovarian cancer. *J. Clin. Oncol. Off. J. Am. Soc. Clin. Oncol.* **19**, 1266–1274 (2001).

15. Cerullo, M. *et al.* Assessing the Financial Burden Associated With Treatment Options for Resectable Pancreatic Cancer: *Ann. Surg.* **267**, 544–551 (2018).

16. O’Neill, C. B. *et al.* Costs and trends in pancreatic cancer treatment. *Cancer* **118**, 5132–5139 (2012).

17. Attard, C. L., Brown, S., Alloul, K. & Moore, M. J. Cost-effectiveness of folfirinox for first-line treatment of metastatic pancreatic cancer. *Curr. Oncol. Tor. Ont* **21**, e41-51 (2014).

18. Aballéa, S. *et al.* Cost-effectiveness analysis of oxaliplatin compared with 5-fluorouracil/leucovorin in adjuvant treatment of stage III colon cancer in the US. *Cancer* **109**, 1082–1089 (2007).

19. Michelakos, T. *et al.* Predictors of Resectability and Survival in Patients With Borderline and Locally Advanced Pancreatic Cancer who Underwent Neoadjuvant Treatment With FOLFIRINOX: *Ann. Surg.* **269**, 733–740 (2019).

20. Blazer, M. *et al.* Neoadjuvant Modified (m) FOLFIRINOX for Locally Advanced Unresectable (LAPC) and Borderline Resectable (BRPC) Adenocarcinoma of the Pancreas. *Ann. Surg. Oncol.* **22**, 1153–1159 (2015).

21. Faris, J. E. *et al.* FOLFIRINOX in locally advanced pancreatic cancer: the Massachusetts General Hospital Cancer Center experience. *The Oncologist* **18**, 543–548 (2013).

22. Christians, K. K. *et al.* Neoadjuvant FOLFIRINOX for Borderline Resectable Pancreas Cancer: A New Treatment Paradigm? *The Oncologist* **19**, 266–274 (2014).

23. Nipp, R. D. *et al.* Predictors of Early Mortality After Surgical Resection of Pancreatic Adenocarcinoma in the Era of Neoadjuvant Treatment. *Pancreas* **46**, 183–189 (2017).

24. Conroy, T. *et al.* FOLFIRINOX or Gemcitabine as Adjuvant Therapy for Pancreatic Cancer. *N. Engl. J. Med.* **379**, 2395–2406 (2018).

25. Sadot, E. *et al.* FOLFIRINOX Induction Therapy for Stage 3 Pancreatic Adenocarcinoma. *Ann. Surg. Oncol.* **22**, 3512–3521 (2015).

26. Bilir, S. P. *et al.* Economic Burden of Toxicities Associated with Treating Metastatic Melanoma in the United States. *Am. Health Drug Benefits* **9**, 203–213 (2016).

27. Caggiano, V., Weiss, R. V., Rickert, T. S. & Linde-Zwirble, W. T. Incidence, cost, and mortality of neutropenia hospitalization associated with chemotherapy. *Cancer* **103**, 1916–1924 (2005).

28. Goldstein, D. A. *et al.* First- and second-line bevacizumab in addition to chemotherapy for metastatic colorectal cancer: a United States-based cost-effectiveness analysis. *J. Clin. Oncol. Off. J. Am. Soc. Clin. Oncol.* **33**, 1112–1118 (2015).

29. Kim, S. S. *et al.* Preoperative FOLFIRINOX for borderline resectable pancreatic cancer: Is radiation necessary in the modern era of chemotherapy?: Preop FOLFIRINOX for Pancreatic Cancer. *J. Surg. Oncol.* **114**, 587–596 (2016).

30. Lloyd, A., Nafees, B., Narewska, J., Dewilde, S. & Watkins, J. Health state utilities for metastatic breast cancer. *Br. J. Cancer* **95**, 683–690 (2006).

31. Gould, M. K., Dembitzer, A. D., Sanders, G. D. & Garber, A. M. Low-molecular-weight heparins compared with unfractionated heparin for treatment of acute deep venous thrombosis. A cost-effectiveness analysis. *Ann. Intern. Med.* **130**, 789–799 (1999).

33. Reni, M. *et al.* Safety and efficacy of preoperative or postoperative chemotherapy for resectable pancreatic adenocarcinoma (PACT-15): a randomised, open-label, phase 2–3 trial. *Lancet Gastroenterol. Hepatol.* **3**, 413–423 (2018).

34. Lazzaro, C. *et al.* An Italian cost-effectiveness analysis of paclitaxel albumin (nab-paclitaxel) + gemcitabine vs gemcitabine alone for metastatic pancreatic cancer patients: the APICE study. *Expert Rev. Pharmacoecon. Outcomes Res.* **18**, 435–446 (2018).
